# Feeding a Mixture of Choline Forms during Lactation Improves Offspring Growth and Maternal Lymphocyte Response to Ex Vivo Immune Challenges

**DOI:** 10.3390/nu9070713

**Published:** 2017-07-07

**Authors:** Erin D. Lewis, Caroline Richard, Susan Goruk, Emily Wadge, Jonathan M. Curtis, René L. Jacobs, Catherine J. Field

**Affiliations:** 1 Department of Agricultural, Food and Nutritional Science, University of Alberta, Edmonton, AB T6G 2E1, Canada; Erin.Lewis@tufts.edu (E.D.L.); cr5@ualberta.ca (C.R.); sgoruk@ualberta.ca (S.G.); wadge@ualberta.ca (E.W.); jcurtis1@ualberta.ca (J.M.C.); rjacobs@ualberta.ca (R.L.J.); 2 Jean Mayer USDA Human Nutrition Center on Aging, Tufts University, Boston, MA 02111, USA

**Keywords:** phosphatidylcholine, glycerophosphocholine, immunology, spleen, mesenteric lymph nodes

## Abstract

Study objectives were to examine the impact of feeding a mixture of choline forms, or a diet high in glycerophosphocholine (GPC) on maternal immune function and offspring growth during lactation. Lactating Sprague-Dawley rat dams (*n* = 6/diet) were randomized to one of three diets, providing 1 g/kg total choline: Control (100% free choline (FC)), Mixed Choline (MC; 50% phosphatidylcholine (PC), 25% FC, 25% GPC), or High GPC (HGPC; 75% GPC, 12.5% PC, 12.5% FC). At 3 weeks, cell phenotypes and cytokine production with Concanavalin A (ConA)-or lipopolysaccharide (LPS)-stimulated splenocytes and mesenteric lymphocytes were measured. Feeding MC or HGPC diets improved pups’ growth compared to Control (+22% body weight, *p* < 0.05). In spleen, MC-and HGPC-fed dams had higher proportions of cytotoxic (CD8+) T cells expressing CD27, CD71 and CD127, total B cells (CD45RA+) and dendritic cells (OX6+OX62+), and produced less IL-6 and IFN-γ after ConA than Control-fed dams (*p* < 0.05). MC and HGPC LPS-stimulated splenocytes produced less IL-1β and IL-6 than Control. ConA-stimulated mesenteric lymphocytes from MC and HGPC dams produced more IL-2 and IFN-γ than Control (*p* < 0.05). In summary, feeding a mixture of choline forms during lactation improved offspring growth and resulted in a more efficient maternal immune response following mitogenic immune challenge.

## 1. Introduction

Choline is an essential micronutrient required in the maternal diet during lactation to ensure adequate choline concentrations in breast milk, support offspring growth [1,2] and maintain maternal immune function [1] and intestinal health [3]. To enable breast milk to meet the demand by the infant to support developing tissues, maternal dietary choline requirements increase, from 425 mg/day for non-pregnant, non-lactating women to 550 mg/day for lactating women. Despite the increased demands during lactation, epidemiological data suggests that many women during this key developmental period are not meeting daily recommendations [4]. We have previously demonstrated that an exogenous source of choline is essential in the maternal diet during lactation for optimal immune function of the mother [1] and the development of the immune system in their offspring [2]. In human populations [5,6], including women during lactation [4], dietary choline is consumed as a variety of forms, primarily as lipid-soluble phosphatidylcholine (PC) (46%), and water-soluble free choline (23%) and glycerophosphocholine (GPC) (19%). In contrast, most commercial rodent diets contain choline in the form of free choline, as a choline salt, based on American Institute of Nutrition (AIN) minimum recommendations [7]. Indeed, given their structural differences, metabolic differences exist between these common dietary forms of choline [8], which may contribute differently to the functions of choline.

Choline is the precursor for a variety components of the immune system including PC and sphingomyelin as structural cellular components, synthesis of acetylcholine [9] and methyl-group donation necessary for proliferation [10]. We have previously demonstrated that providing choline in the maternal diet during the lactation period as PC, instead of free choline, alters immune function in both lactating dams and offspring [11]. Feeding PC in the maternal diet during lactation improves the response of the dam’s peripheral and gut-associated immune systems [12] and promotes maturation of the immune system in suckled offspring [11]. GPC is another commonly consumed form of choline, found primarily in dairy products including milk and cheese [13,14]. In addition to its role as an osmolyte in the kidney [15], GPC may serve as a cellular structural component [16] and precursor for immunomodulatory compounds including platelet-activating factor (reviewed in [17]). However, the potential immune regulating effects of GPC have not been investigated.

This evidence suggests that the different dietary forms of choline in the maternal diet do not confer the same immunological benefits, yet this is not representative of the mixture of choline forms consumed during pregnancy and lactation [4]. In offspring, providing a mixture of choline forms in the maternal diet during lactation resulted in a more mature lymphocyte population which was beneficial for T cell function [18]. However, the effects of modulating the forms of choline in the maternal diet on dam’s immune function have not been assessed. Therefore, the objectives of the current study were to examine parameters of maternal immune function and offspring growth during lactation when dams are fed the recommended amount of total choline (1 g/kg, AIN recommendations [7]) as (1) a mixture of choline forms (50% PC, 25% FC, 25% GPC), and (2) a diet providing choline primarily as GPC (75% GPC, 12.5% PC, 12.5% FC), compared to a diet comprised of 100% FC.

## 2. Materials and Methods

### 2.1. Animals and Diets

Female Sprague-Dawley rats at 14 days gestation (*n* = 18) were obtained from Charles River Laboratories (Montreal, QC, Canada). Dams were fed standard rat chow (Lab diet 5001; PMI Nutrition International, Brentwood, MO, USA) throughout gestation, then randomized to one of three experimental diets (Table 1) 24–48 h prior to parturition.

Diets were fed ad libitum throughout lactation to the end of the study at 21 days postnatal. Animals had free access to food throughout each 24-h period and feed cups were refilled every 2–3 days. The three experimental diets all contained 1 g of choline/kg of diet, were isocaloric and isonitrogenous and differed only in the form of choline provided (Table 1): Control diet (1 g/kg of choline as 100% FC; *n* = 6), Mixed Choline diet (MC) (1 g/kg of choline as 50% PC, 25% FC, 25% GPC; *n* = 6), High GPC diet (HGPC) (1 g/kg of choline as 75% GPC, 12.5% PC, 12.5% FC; *n* = 6). As PC provided some lipid, the lipid content and composition of the experimental diets were adjusted to ensure that the diets contained similar fat content (% *w*/*w*). The fatty acid composition of the experimental diets was analyzed by gas liquid chromatography as previously described [20] (Table S1).

At birth, litters were standardized to ten pups (5 males and 5 females when possible) per dam. Dietary intake and body weight were recorded regularly throughout the study period. The protocol was reviewed and approved by the Committee of Animal Policy and Welfare of the Faculty of Agricultural, Life and Environmental Sciences at the University of Alberta, Edmonton, AB, Canada (AUP00000125_REN5).

### 2.2. Tissue Collection

Twenty-one days after parturition, dams and two offspring (one male and one female) per dam were weighed and euthanized by CO_2_ asphyxiation in the morning hours. From the dams, spleens were collected aseptically, weighed, and immune cells were isolated for further processing (see Immune cell isolation section below). Mesenteric lymph nodes were collected aseptically and immune cells were isolated for further processing (see Immune cell isolation section below).

### 2.3. Choline Metabolite Analyses of Dam’s Splenocytes and Offspring Stomach Content

Stomach contents of pups were analyzed to reflect the choline concentration in the pup’s diet during lactation. Splenocytes and frozen stomach content were extracted using a modified Bligh and Dyer method that has been previously described in [21,22]. Extracts were quantified for all significant choline-containing metabolites and total choline content by HILIC liquid chromatography-tandem mass spectrometry (LC-MS/MS) using an Agilent 1200 series HPLC system (Agilent Technologies, Palo Alto, CA, USA) coupled to a 3200 QTRAP mass spectrometer (AB SCIEX, Concord, ON, Canada). The choline content in splenocytes was adjusted for protein content, which was measured using a commercial bicinchoninic acid (BCA) assay kit (Thermo Fisher Scientific, Edmonton, AB, Canada) according to the manufacturer’s instructions.

### 2.4. Immune Cell Isolation

Isolation of immune cells from spleen and mesenteric lymph nodes have been previously described [23]. Briefly, single cell suspensions were obtained by disrupting tissue through a nylon mesh screen in sterile Krebs–Ringer HEPES buffer with bovine serum albumin (5 g/L; Sigma-Aldrich Canada Ltd., Oakville, ON, Canada). Ammonium chloride lysis buffer (155 mM NH_4_Cl, 0.1 mM EDTA, 10 mM KHCO_3_; Fisher Scientific, Edmonton, AB, Canada) was used to lyse erythrocytes. Cells were washed then re-suspended in complete culture medium (RPMI 1640 media; Life Technologies, Burlington, ON, Canada), supplemented with 5% (*v*/*v*) heat-inactivated fetal calf serum, 25 mM HEPES, 2.5 mM 2-mercaptoethanol and 1% antibiotic/antimycotic (pH 7.4; Fisher Scientific, Edmonton, AB, Canada). Prior to ex vivo analyses, a haemocytometer was used to count live cells using trypan blue dye exclusion (Sigma-Aldrich, Oakville, ON, Canada) to assess cell viability and was >90% for all treatment groups. All cell suspensions were then diluted to 1.25 × 10^6^ cells/mL.

### 2.5. Immune Cell PhenotyPe Analysis

Immune cells from spleen and mesenteric lymph nodes were isolated as previously described [1,23]. Immune cell subsets present in freshly isolated splenocytes and mesenteric lymph node cells were identified by direct immunofluorescence assay, as previously described [23,24]. The use of four-color flow cytometry allowed identification of the following combinations of surface molecules in splenocytes: CD28/CD3/CD8/CD4, CD25/CD152/CD8/CD4, CD25/CD127/CD8/CD4, CD27/CD8/CD4, CD27/OX12/OX6/CD45ra, CD71/CD8/CD4, OX12/OX6/CD80, CD86/CD80/CD45RA, CD68/CD284/CD11b/c, OX62/CD25/OX6, CD161/OX62/CD3, IgG/IgM, IgA. The following combinations of surface molecules were used for mesenteric lymph node cells: CD3/CD45RA, CD28/CD3/CD4/CD8, CD25/CD152/CD8/CD4, CD25/CD127/CD8/CD4, CD27/CD8/CD4, CD71/CD8/4, IgG/IgM, IgA. All antibodies with the exception of IgG, IgM and OX6 (BD Biosciences, Mississauga, ON, Canada) were purchased from Cedarlane Laboratories, (Burlington, ON, Canada). After incubation, cells were washed and fixed in paraformaldehyde (10 g/L; Thermo Fisher Scientific, Edmonton, AB, Canada) in phosphate-buffered saline. All of the samples were acquired within 72 h of preparation by flow cytometry (FACSCalibur; Becton Dickinson, San Jose, CA, USA) according to the relative fluorescence intensity determined using Kaluza Software (Beckman Coulter, Mississauga, ON, Canada).

### 2.6. Ex Vivo Cytokine Production by Mitogen-Stimulated Cells

The measurement of the production of cytokines by mitogen-stimulated cells in spleen and mesenteric lymph nodes have been previously described [25]. Briefly, cells (1.25 × 106 cells/mL) were cultured in 3 mL RMPI-1640 medium, as described above, for 48 h at 37 °C and 5% CO_2_ without mitogen (unstimulated) or with mitogen ConA (5 µg/mL; MP Biomedicals, Montreal, QC, Canada), lipopolysaccharide (LPS, 100 µg/mL; Sigma-Aldrich, Oakville, ON, Canada). ConA is a polyclonal T-cell stimulant and LPS activates the antigen-presenting cell population, including dendritic cells, macrophages and B cells by binding to their Toll-like receptor (CD284). After incubation, cells were centrifuged for 10 min at 1000 rpm and supernatants collected and stored at −80 °C until analyses. Concentrations of cytokines interleukin (IL)-1β, IL-2, IL-6, IL-10, tumor-necrosis factor-α (TNF-α), and interferon-γ (IFN-γ) were measured by commercial ELISA kits according to the manufacturer’s instructions and as previously described (26). The detection limits for all cytokines were 15.6–4000 pg/mL, except for IFN-γ in which the detection limit was 9.8–2500 pg/mL (R&D Systems, Minneapolis, MN, USA). Cytokine concentrations were quantified using a microplate reader (SpectraMax 190; Molecular Devices, Sunnyvale, CA, USA) and all measurements were conducted in duplicates, with coefficient of variation (CV) <10%. The amount of IL-2 in the media after LPS stimulation was below detection levels. IL-1β was only measured in the supernatant of LPS stimulated cells.

### 2.7. Statistical Analyses

Data are reported as mean ± standard error of the mean (SEM) unless indicated otherwise. The study was powered to assess significant changes in immune function (i.e., ex vivo cytokine production as the primary outcome). Data were analyzed using one-way ANOVA in SAS (v9.4, Cary, NC, USA) with diet as the main effect. In cases where a significant main effect of diet was found, post hoc analysis was performed using the Duncan adjustment to determine differences between diet groups. Variables not normally distributed were log-10 transformed prior to statistical analysis. Differences at *p* ≤ 0.05 (two-sided) were considered significant.

## 3. Results

### 3.1. AnthroPometric Characteristics and Daily Food Intake

At 21 days postnatal, dams fed the MC or HGPC diets had higher average pup weight than pups from dams fed the Control diet (Table 2). There were no significant differences in organ weights, intestinal length or relative number of splenocytes (number of splenocytes/g spleen) amongst diet groups (Table 2). Mean daily food intake of the dams in each group for the duration of the lactation period (21 days) was 47 ± 1 g/day (mean of all the dams, *n* = 18) and did not differ amongst diet groups (Table 2).

### 3.2. Choline Metabolites in Pup’s Stomach Content and Dams’s SPleen

Total choline concentration in pups’ stomach content was not significantly different among groups (Table S2). However, compared to the pups from the Control-fed dams, the relative contribution of PC to total choline was higher in stomach content of pups from MC-fed dams (*p* < 0.01). Moreover, pups from HGPC-fed dams had a higher proportion of GPC, and lower proportion of free choline and phosphocholine in stomach content compared to pups from Control-fed dams (all *p* < 0.05) (Table S2).

Mean concentration of total choline in splenocytes was not different amongst diet groups (Table 3). The major choline-containing metabolite found in spleen was PC, and was not significantly different among diet groups. Interestingly, splenocytes from dams fed the MC and HPC diets had an approximately 3-fold higher concentration of lysoPC compared to Control-fed dams (*p* < 0.05) (Table 3). There was also a higher concentration of phosphocholine in spleen of MC-fed dams compared to both Control and HGPC-fed dams (*p* < 0.05) (Table 3). There was a trend (*p* = 0.084) towards higher concentration of GPC in splenocytes from dams fed the HGPC diet compared to dams fed the MC or Control diets.

### 3.3. Splenocyte Phenotypes

There was no significant difference in the proportion of total T cells (CD3+) among diet groups, or in the absolute number of T cells (total CD3+, CD3+CD4+ and CD3+CD8+) (Table S3). However, within the T cell population, there was a significantly lower proportion of the subset of T helper cells (Th, CD4+) in spleen of HGPC-fed dams compared to Control-fed dams (Table 4). Within the CD8+ (cytotoxic) T cell subset, HGPC- and MC-fed dams had a significantly higher proportion of CD8+ expressing CD27 (TNF receptor) and CD127 compared to Control-fed dams (all *p* < 0.05) (Table 4). MC-fed dams also had a higher percentage of CD8+ T cells expressing CD71 (transferrin receptor) as well as a higher proportion of CD4+ T cells expressing CD28 (co-stimulatory molecule) compared to Control-fed dams (all *p* < 0.05) (Table 3). MC-and HGPC-fed dams both had higher percentage of dendritic cells (OX62+OX6+) and total B cells (CD45RA+) and a lower proportion of activated B cells (CD80+CD45RA+) and IgG+ cells in spleen compared to Control-fed dams (all *p* < 0.05) (Table 4). There were few differences in splenocyte phenotypes between MC-and HGPC-fed dams, with only a higher proportion of macrophages (CD68+) expressing the toll-like receptor-4 (TLR-4; CD284+) in HGPC-fed dams compared to MC-fed dams (*p* < 0.05) (Table 4).

### 3.4. Mesenteric Lymphocyte Phenotypes

Diet did not have a large effect on mesenteric lymphocyte phenotypes (Table 5 and Table S3). Overall, there were no changes in the proportion (Table 5) or absolute number (Table S3) of total T cells (CD3+), T cell subsets (CD4+ and CD8+), or total B cells (CD45RA+). HGPC-fed dams had a significantly higher percentage of CD8+ T cells expressing CD152 (cytotoxic T-lymphocyte-associated protein 4, CTLA-4) compared to FC-fed dams (*p* < 0.05) (Table 4). Within the B cell population, there was a significantly lower proportion of IgA+ cells from dams fed the MC-or HGPC-fed diets compared to dams fed the Control diet (*p* < 0.05). Similarly, MC-fed dams had a lower proportion of IgG+ cells in mesenteric lymph nodes compared dams fed the Control diet (Table 5).

### 3.5. Ex Vivo Cytokine Production after Stimulation

**Figure 1:**
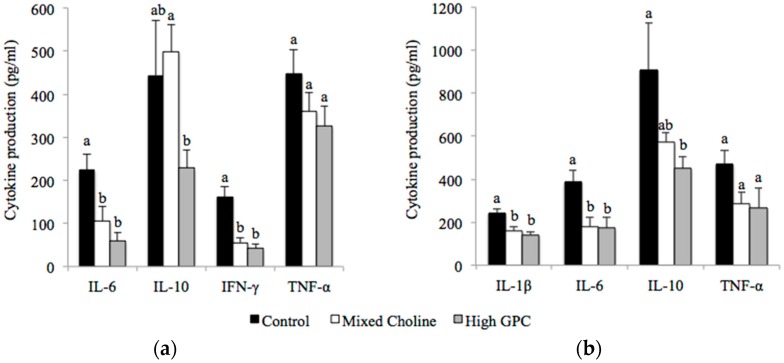
Cytokine production by splenocytes after ex vivo stimulation with Concanavalin A (ConA) (**a**) or lipopolysaccharide (LPS); (**b**) from lactating dams fed Control (100% FC) (*n* = 6), Mixed Choline (MC; 50% PC, 25% FC, 25% GPC) (*n* = 6) or High GPC (HGPC; 75% GPC, 12.5% PC, 12.5% FC) (*n* = 6) diets. FC, free choline; GPC, glycerophosphocholine; PC, phosphatidylcholine. Values are presented as mean ± SEM. *p* value of the main effect of diet analyzed by one-way ANOVA. Multiple comparisons between diet groups have been performed with Duncan adjustment. Means within a row that do not share a common superscript letters a, b are significantly different (*p* < 0.05).

In spleen, there was no difference in IL-2 (Figure S1) or TNF-α production following ex vivo stimulation with ConA among diet groups (Figure 1a). However, splenocytes from MC or HGPC-fed dams produced significantly less IL-6 and IFN-γ than splenocytes from Control-fed dams after ConA stimulation (Figure 1A) (*p* < 0.05). The significantly lower production of IFN-γ resulted in a significantly lower ratio of IFN-γ to IL-2 from splenocytes of MC dams (0.0048) and HGPC dams (0.0042) compared to Control dams (0.014) (*p* < 0.05). The only difference between MC and HGPC dams was lower IL-10 production by splenocytes from HGPC-fed dams following ConA stimulation. Following LPS stimulation, splenocytes from MC-and HGPC-fed dams produced significantly less IL-1β, IL-6 and IL-10 than Control-fed dams (*p* < 0.05) (Figure 1b). TNF-α production by splenocytes was not affected by diet following LPS stimulation.

**Figure 2:**
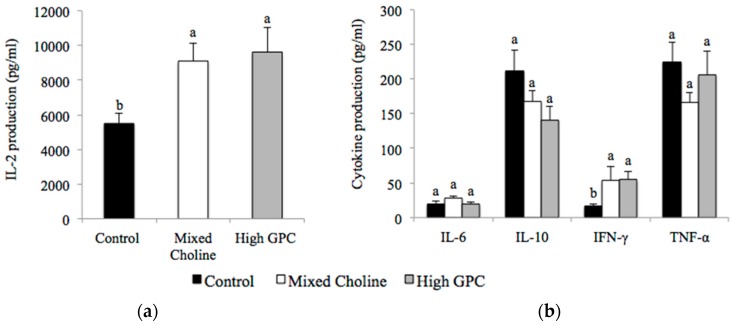
IL-2 production (**a**) and cytokine production; (**b**) by mesenteric lymphocytes after ex vivo stimulation with Concanavalin A (ConA) from lactating dams fed Control (100% FC) (*n* = 6), Mixed Choline (MC; 50% PC, 25% FC, 25% GPC) (*n* = 6) or High GPC (HGPC; 75% GPC, 12.5% PC, 12.5% FC) (*n* = 6) diets. FC, free choline; GPC, glycerophosphocholine; PC, phosphatidylcholine. Values are presented as mean ± SEM. *p* value of the main effect of diet analyzed by one-way ANOVA. Multiple comparisons between diet groups have been performed with Duncan adjustment. Means within a row that do not share a common superscript letters a, b are significantly different (*p* < 0.05).

In mesenteric lymph nodes, dams fed MC or HGPC diets produced significantly more IL-2 (Figure 2a) and IFN-γ (Figure 2b) compared to dams fed the Control diet (*p* < 0.05). There was no difference in IL-6, IL-10 and TNF-α production or the ratio of IFN-γ to IL-2 between diet groups following ConA stimulation (Figure 2b). There was no difference in LPS response by mesenteric lymphocytes between diet groups (Table S4).

## 4. Discussion

The current study demonstrates for the first time that feeding the same amount of total choline (1 g/kg diet) but provided as a mixture of choline forms, either representative of human dietary consumption (50% PC, 25% FC, 25% GPC) [4] or high in GPC, significantly altered lactating dams’ function of lymphocytes in the spleen (peripheral) and mesenteric lymph nodes (gut-associated), compared to providing choline only as FC. Furthermore, providing a mixture of choline forms, or primarily as GPC, in the maternal diet also improved offspring growth at the end of the 3-week suckling period. Interestingly, we previously observed that feeding choline only as PC in the maternal diet during lactation did not affect offspring growth compared to feeding only FC [11]. Choline metabolite concentrations in stomach content (representative of offspring diet) may be associated with offspring growth. Our results suggest that higher concentrations of choline as PC or GPC, while reciprocally having less free choline and phosphocholine in the offsprings’ diet, is associated with improved growth in the offspring during a critical period of development. We observed a 12% reduction in the proportion of phosphocholine, a water soluble form of choline that comprises a large majority of rat, human and bovine milk [8]. Phosphocholine is an intermediate in the CDP-choline pathway, formed when free choline is phosphorylated via enzyme choline kinase; therefore, the reduction in the proportion of phosphocholine is likely attributed to the decrease in precursor, FC. Richard et al. (2017) has further described the effects of altering the forms of choline in the maternal diet on offspring stomach content [18]. At this time, the potential consequences of altering the forms of choline in the offspring’s diet, such as reducing the proportion of choline from phosphocholine, are unknown. Previous studies [11,26] that have observed differences in choline forms have proposed that future studies should address the role of the difference forms of choline in the maternal and offspring diet, and the implications on offspring health.

Feeding a MC diet or a diet high in GPC altered the ex vivo response of splenocytes following stimulation with ConA, a non-specific polyclonal T cell mitogen and a bacterial antigen, LPS. Dams fed the MC or HGPC diets produced approximately 70% less IFN-γ and approximately 62% less IL-6 compared to FC-fed dams following ConA stimulation. IFN-γ [27] and IL-6 [28] are T-helper 1 (Th1) cytokines involved in mediating both pro- and anti-inflammatory processes. Notably, IL-2 production was not different among diet groups, suggesting that proliferation was not altered by choline source. The lower IFN-γ in the context of same IL-2 and resulting lower IFN-γ to IL-2 ratio, suggests that splenocytes produce a lower Th1 response. This is suggests a more efficient immune response. The MC- or HGPC-fed dams do not need to produce as many cytokines in order to maintain the same proliferative response (IL-2 production) as FC-fed dams in response to a T cell mitogen. Lower production of cytokines following mitogenic challenge, while maintaining the same proliferative state (IL-2 production), suggests that splenocytes from MC-or HGPC-fed dams are more efficient at mediating an inflammatory response to immune challenge. In response to LPS stimulation, splenocytes from MC- or HGPC-fed dams produced significantly less IL-1β and IL-6 compared to FC-fed dams. LPS is a bacterial antigen that activates antigen presenting cells including B cells, macrophages and dendritic cells, when it binds to the TLR-4 (CD284) present on the surface of these cells. One notable difference between the experimental mixed choline diet groups in response to LPS stimulation is that HGPC-fed dams produced 55% less IL-10 compared to MC-fed dams. IL-10 is classically categorized as an anti-inflammatory cytokine, and meditator of other pro-inflammatory cytokines that can be produced by a variety of immune cells including CD8+ T cell subsets, macrophages and B cells [29]. The lower response of key cytokines, IL-6 and IL-1β, following bacterial challenge from splenocytes from MC-or HGPC-fed dams requires further study as it is possible lower production of cytokines may indicate a less efficient response to bacterial challenge. There was no difference in the proportion of CD45RA+ cells (total B cells) but the current study did not look at the maturation of functional indices on these B cells. Changes in these may explain the difference in the ex vivo response. As this is an experimental animal model, future studies will be needed to confirm the biological significance of ex vivo measurements. Furthermore, the effects of choline in the maternal diet on systemic inflammation could be examined by assessing in vivo measures of immune function, including systemic concentrations of cytokines or soluble cytokine receptors or immunoglobulins.

Response to ex vivo stimulation may be partially explained by the immune cell phenotypes present in the spleen. MC- or HGPC-fed dams had greater proportions of the cytotoxic (CD8+) T cell population expressing CD27, a marker of T cell memory. In humans, memory cytotoxic T cells have been demonstrated to be involved in decreased production of pro-inflammatory cytokines [30]. Furthermore, greater proportions of the cytotoxic (CD8+) T cell population in spleen of MC- or HGPC-fed dams expressed markers of early activation (CD71) and differentiation and maturation (CD127). The transferrin receptor, CD71, is a marker of cellular iron uptake and expressed on activated and proliferating T cells [31], suggesting that a more activated lymphocyte phenotype may be more efficient at responding to immune challenge. Compared to FC-fed dams, dams fed the MC or HGPC diets also had greater proportions of accessory cells including dendritic cells (OX2+OX6+) and total B cells (CD45RA+), and HGPC-fed dams had a higher proportion of macrophages (CD68+) expressing the TLR-4, the receptor that binds LPS. The higher proportion of antigen-presenting cells including B cells, dendritic cells and macrophages expressing TLR-4 in spleen may aid in mediating the inflammatory response by the activated T cell population while maintaining a similar proliferative response. Collectively, this data suggests that a maternal diet providing a mixture of choline forms, or high in GPC, activates T and accessory cells which may facilitate a more efficient immune response following mitogen stimulation, compared to providing only FC. This is consistent with a previous study in which feeding choline only as PC in the maternal diet promotes a more efficient response by antigen-presenting cells in offspring, compared to feeding choline as FC [11]. It is possible that providing different dietary forms of choline, both PC and GPC, compared to the standard FC diet, may be advantageous to the development and function of antigen presenting cells in both dam and offspring. In the current study, feeding a mixed choline diet, or diet high in GPC resulted in a higher concentration of lysoPC in splenocytes, compared to feeding a diet comprised of only FC. This suggests that altering the choline composition of the diet is capable of altering the membrane composition of spleen lymphocytes, which might contribute to differences in function. The mechanisms involved have not been elucidated, but an in vitro study in splenocytes demonstrated that providing lysoPC, the form of PC readily taken up by cells in vitro, increases cellular proliferation and activation when stimulated [11]. We did not measure the composition of mesenteric lymphocytes, however it would be of interest in future studies to confirm that other immune tissues are also modulated similarly by the form of choline in the diet.

In addition to alteration of peripheral immune functions, the forms of choline in the maternal diet alter gut-associated immune functions. Mesenteric lymph nodes consist primarily of T and B lymphocytes and are a critical part of the gut-associated immune system that serves as the first line of defense against antigen or pathogen exposure via the oral route (reviewed in [32]). The activation of mesenteric lymphocytes promotes production of cytokines and other inflammatory mediators which induce other immune cells to respond to invading pathogens [33]. The current study demonstrates that the forms of choline in the maternal diet alter the ability of mesenteric lymphocytes to respond to immune challenge, which may have implications on host defense. Notably, after stimulation with a ConA, mesenteric lymphocytes from MC- or HGPC-fed dams produced 1.6-and 1.8-fold more IL-2 compared to FC-fed dams, respectively. This is different than what occurs in spleen, in which IL-2 production was not different amongst diet groups. This is consistent with our previous findings in which feeding only PC, compared to FC, resulted in higher production of IL-2 with ConA stimulation in mesenteric lymph nodes of lactating dams [12]. IL-2 is secreted primarily by activated helper (CD4+) and cytotoxic (CD8+) T cells and plays a critical role in inducing cellular proliferation and differentiation of antigen-activated T cells into different T cell subsets including regulatory and memory T cells [34,35]. Differentiation of regulatory T cells mediate tolerance [36,37], which is particularly critical in the gut-associated immune system. Providing PC and GPC in the maternal diet may be critical for inducing IL-2 production and to the response to mitogen stimulation by mesenteric lymphocytes which may aid in host defenses. A more robust immune response likely explains higher production IFN-y, with MC-or HGPC-fed dams producing approximately 3.5-fold more IFN-γ compared to FC-fed dams. IFN-γ is involved in the skewing of a Th1 phenotype and assists in the maintenance of humoral (antibody-mediated) responses and mounting appropriate responses for the elimination of viral and bacterial pathogens [38]. IL-2 has been shown to induce the production of IFN-y [39], therefore it is not surprising that higher IL-2 production upon stimulation is accompanied by higher IFN-y production. The response to LPS by mesenteric lymphocytes was not affected by the forms of choline in the maternal diet. Interestingly, feeding a MC or HGPC diet had little effect on mesenteric lymphocyte phenotypes. This suggests that providing choline as PC or GPC may have direct effects on the ability of lymphocytes to produce cytokines.

Feeding a diet containing a mixture of choline forms (PC, GPC and FC) or primarily as GPC resulted in similar changes in maternal immune function. This highlights a possible metabolic interrelationship between choline metabolites. PC may be used to produce GPC, first by conversion to lysoPC, via the action of phospholipases A_1_ and A_2_. GPC may also be converted to PC (via the Kennedy pathway) in splenocytes. We believe that the responses of the two diets may be similar due to the fact that there was similar choline metabolite distribution in the splenocytes, the immune tissue of interest. As hypothesized in our previous study [11], it is possible that other dietary choline metabolites could contribute to the changes in immune function observed. It is likely that a source of GPC in the diet may be mediating immune functions, independent of the amount of dietary PC. In vitro studies in rodent and bovine gonadal tissue have demonstrated that oxidized GPC, lysoglycerophosphocholine, inhibits IL-2 production and T cell proliferation [40]. However, consistent with our observations, Tokes et al., (2015) reported in vivo that administration of GPC attenuated intestinal superoxide production after ischaemia-reperfusion injury, suggesting a possible anti-inflammatory role of GPC [41]. Our study in lactating dams and offspring [18] was the first to specifically look, in an experimental model, of providing GPC, a major choline metabolite found in dairy foods (particularly milk), in the diet. As a supply of GPC appears to be important, future studies should be designed to elucidate the immunomodulatory actions and mechanisms of different proportions of GPC in mixtures containing the other choline containing molecules.

## 5. Conclusions

In summary, the results of the present study demonstrate that feeding diets containing mixtures of choline forms to lactating dams improved offspring growth during the suckling period. Providing a mixture of choline forms, as PC, FC and GPC, or primarily as GPC, resulted in a better proliferative response to a T cell mitogen by cells from the gut-associated lymphoid system and a more efficient immune response to T cell mitogen by systemic immune cells. Overall, our results provide evidence that the forms of choline in the maternal diet should be considered when examining maternal and offspring health.

## Figures and Tables

**Table 1:** Composition of experimental diets ^1^.

Component (g/kg Diet)	Control Diet	MC Diet	HGPC Diet
Casein	270	270	270
Starch	240	240	240
Sucrose	126	126	126
Vitamin mix (AIN-93-Vx) ^2^	19	19	19
Mineral mix ^3^	50	50	50
Calcium phosphate dibasic	3.4	3.4	3.4
Inositol	6.3	6.3	6.3
Cellulose	80.0	80.0	80.0
l-cysteine	1.8	1.8	1.8
Fat mixture			
Canola oil	40	31	39
Olive oil	13	13.8	8.5
Vegetable oil	14	14	14.3
Corn oil	73	65	71.9
Sunflower oil	2	5	2.8
Flax seed oil	2	2	2
Hydrogenated canola oil	56	57.5	56
DHAsco	1.5	1.5	1.5
ARAsco	1.5	1.5	1.5
Choline mixture (providing 1 g of total choline/kg diet)			
Soy lecithin (PC)	0	13.1	3.2
Choline bitartrate	2.1	0.6	0.3
Glycerophosphocholine	0.0	0.6	1.9

GPC, glycerophosphocholine; LysoPC, lysophosphatidylcholine; PC, phosphatidylcholine. ^1^ All ingredients were purchased from Harlan Teklad (Indianapolis, IN, USA), with the exception of the dietary oils that were all purchased from Safeway (Edmonton, AB, Canada). The hydrogenated canola oil was donated by Richardson Oilseed Limited (Lethbridge, AB, Canada) and ARAsco and DHAsco were donated by DSM (Nutritional Products, Columbia, MD, USA); ^2^ AIN-93-VX Vitamin mix [7]; ^3^ Bernhart–Tomarelli salt mixture [19].

**Table 2:** Anthropometric data of lactating dams fed Control (100% FC), Mixed Choline (MC; 50% PC, 25% FC, 25% GPC) or High GPC (HGPC; 75% GPC, 12.5% PC, 12.5% FC) diets at the end of study period, 21 days postnatal.

	Control (*n* = 6)	MC (*n* = 6)	HGPC (*n* = 6)	*p* Value
Body weight (g)	302 ± 11	322 ± 9	318 ± 5	0.284
Spleen weight (g)	0.75 ± 0.02	0.81 ± 0.05	0.79 ± 0.02	0.385
Splenocytes/g spleen (×10^6^)	275 ± 14.9	220 ± 12.1	296 ± 20.3	0.086
Mesenteric lymphocytes/animal (×10^6^)	6.3 ± 0.8	8.0 ± 0.7	6.3 ± 0.8	0.169
Liver weight (g)	14.6 ± 1.4	16.7 ± 0.8	15.1 ± 0.5	0.285
Intestine length (cm)	139 ± 4	145 ± 3	141 ± 2	0.339
Food Intake (g/day)	47 ± 3	46 ± 1	47 ± 1	0.395
Average pup weight (g)	60 ± 3 ^b^	74 ± 3 ^a^	72 ± 3 ^a^	0.007

FC, free choline; GPC, glycerophosphocholine; LysoPC, lysophosphatidylcholine; PC, phosphatidylcholine. Values are presented as mean ± SEM. *p* value of the main effect of diet analyzed by one-way ANOVA. Multiple comparisons between diet groups have been performed with Duncan adjustment. Means within a row that do not share a common superscript letters a, b are significantly different (*p* < 0.05).

**Table 3:** Choline-containing metabolites in splenocytes of lactating dams fed Control (100% FC), Mixed Choline (MC; 50% PC, 25% FC, 25% GPC) or High GPC (HGPC; 75% GPC, 12.5% PC, 12.5% FC) diets.

Choline-Containing Metabolite (µg/mg Protein)	Control (*n* = 6)	MC (*n* = 6)	HGPC (*n* = 6)	*p* Value
PC	19.3 ± 1.6	18.4 ± 1.8	15.4 ± 1.4	0.205
Free choline	0.45 ± 0.04	0.43 ± 0.04	0.56 ± 0.07	0.232
GPC	0.30 ± 0.03	0.27 ± 0.04	0.52 ± 0.13	0.084
LysoPC	0.36 ± 0.04 ^b^	1.10 ± 0.1 ^a^	1.07 ± 0.1 ^a^	0.0001
Sphingomyelin	3.4 ± 0.3	4.1 ± 0.4	4.2 ± 0.3	0.176
Phosphocholine	1.1 ± 0.04 ^b^	1.5 ± 0.04 ^a^	1.3 ± 0.06 ^b^	0.0015
Total choline (µg/mg protein)	4.3 ± 0.2	4.6 ± 0.3	4.3 ± 0.2	0.669

FC, free choline; GPC, glycerophosphocholine; LysoPC, lysophosphatidylcholine; PC, phosphatidylcholine. Values are presented as mean ± SEM. *p* value of the main effect of diet analyzed by one-way ANOVA. Multiple comparisons between diet groups have been performed with Duncan adjustment. Means within a row that do not share a common superscript letters a, b are significantly different (*p* < 0.05).

**Table 4:** Splenocyte phenotypes of lactating dams fed Control (100% FC), Mixed Choline (MC; 50% PC, 25% FC, 25% GPC) or High GPC (HGPC; 75% GPC, 12.5% PC, 12.5% FC) diets.

Cell Phenotype	Control (*n* = 6)	MC (*n* = 6)	HGPC (*n* = 6)	*p* Value
	% of total lymphocytes			
Total CD3+ (T cell)	44.6 ± 1.8	40.9 ± 1.8	36.9 ± 3.5	0.167
	% of CD3+ cells			
CD4+	59.3 ± 1.7 ^a^	57.5 ± 4.6 ^a^	51.1 ± 2.7 ^b^	0.011
CD8+	34.8 ± 0.9	32.1 ± 1.5	36.1 ± 1.7	0.164
	% of CD4+ T cells			
CD27+	73.7 ± 9.5	81.8 ± 2.7	86.3 ± 2.8	0.342
CD28+	84.2 ± 6.1 ^b^	98.9 ± 0.6 ^a^	94.7 ± 1.7 ^ab^	0.035
CD71+	6.9 ± 1.1 ^b^	11.1 ± 1.0 ^a^	13.4 ± 1.2 ^a^	0.041
CD127+	2.9 ± 0.5	2.1 ± 0.2	2.6 ± 0.6	0.558
	% of CD8+ T cells			
CD27+	79.3 ± 8.1 ^b^	98.2 ± 1.3 ^a^	99.7 ± 0.2 ^a^	0.013
CD28+	71.7 ± 6.3	74.1 ± 1.4	69.1 ± 2.4	0.683
CD71+	7.9 ± 0.8 ^b^	13.2 ± 0.9 ^ab^	16.4 ± 3.2 ^a^	0.023
CD127+	3.9 ± 0.9 ^b^	5.1 ± 0.3 ^a^	8.2 ± 1.2 ^a^	0.011
	% of total lymphocytes			
CD68+CD284+	8.3 ± 0.3 ^ab^	7.9 ± 0.2 ^b^	9.0 ± 0.3 ^a^	0.045
OX62+OX6+ (Dendritic cell)	4.4 ± 1.1 ^b^	8.6 ± 0.5 ^a^	9.2 ± 0.6 ^a^	0.001
Total CD45RA+ (B cells)	30.7 ± 1.0 ^b^	40.4 ± 1.3 ^a^	39.1 ± 4.7 ^a^	0.034
CD80+CD45RA+	4.8 ± 0.5 ^a^	2.9 ± 0.2 ^b^	3.7 ± 0.2 ^b^	0.004
IgA+	12.1 ± 0.6	12.1 ± 0.3	13.6 ± 0.6	0.105
IgG+	10.4 ± 1.1 ^a^	5.4 ± 0.8 ^b^	3.2 ± 0.2 ^b^	0.0003
IgM+	49.6 ± 1.5 ^b^	55.2 ± 0.8 ^a^	53.1 ± 1.9 ^ab^	0.045

FC, free choline; GPC, glycerophosphocholine; LysoPC, lysophosphatidylcholine; PC, phosphatidylcholine. Values are presented as mean ± SEM. *p* value of the main effect of diet analyzed by one-way ANOVA. Multiple comparisons between diet groups have been performed with Duncan adjustment. Means within a row that do not share a common superscript letters a, b are significantly different (*p* < 0.05). The following phenotypes not shown in table were not significantly different among groups (mean ± SEM, % of total cells, n = 18): CD4+CD25+, 7.0 ± 0.5; CD8+CD25+, 4.7 ± 0.5; CD4+CD152+, 2.7 ± 1.1; CD8+CD152+, 8.4 ± 1.2; CD3-CD161+, 2.5 ± 0.3; CD3+CD161+, 0.8 ± 0.2; OX6+OX12+, 26.3 ± 1.6; OX6+CD80+, 4.9 ± 0.6; OX12+CD80+, 4.4 ± 0.5; CD27+CD45RA+, 2.8 ± 0.7.

**Table 5:** Mesenteric lymphocyte phenotypes of lactating dams fed Control (100% FC), Mixed Choline (MC; 50% PC, 25% FC, 25% GPC) or High GPC (HGPC; 75% GPC, 12.5% PC, 12.5% FC) diets.

Cell Phenotype	Control (*n* = 6)	MC (*n* = 6)	HGPC (*n* = 6)	*p* Value
% of total lymphocytes				
Total CD3+ (T cell)	57.0 ± 1.6	58.5 ± 1.1	60.3 ± 1.5	0.278
% of CD3+ cells				
CD4+	69.3 ± 0.6	70.1 ± 1.4	66.0 ± 1.4	0.061
CD8+	26.0 ± 1.6	26.5 ± 3.8	22.4 ± 3.7	0.628
% of CD4+ T cells				
CD71+	6.2 ± 0.9	5.5 ± 0.8	6.8 ± 0.6	0.484
CD127+	0.73 ± 0.2	1.5 ± 0.3	2.7 ± 1.2	0.215
CD152+ (CTLA-4+)	1.8 ± 1.1	0.8 ± 0.1	2.4 ± 0.9	0.433
% of CD8+ T cells				
CD71+	7.0 ± 0.7	7.9 ± 1.3	5.7 ± 0.5	0.258
CD127+	1.0 ± 0.2	1.1 ± 0.1	1.9 ± 0.5	0.104
CD152+ (CTLA-4+)	5.7 ± 0.5 ^b^	5.4 ± 0.4 ^ab^	7.9 ± 0.6 ^a^	0.005
% of total lymphocytes				
Total CD45RA+ (B cells)	26.8 ± 1.3	36.1 ± 1.3	33.7 ± 0.1	0.554
IgA+	4.3 ± 0.6 ^a^	1.6 ± 0.1 ^b^	2.3 ± 0.3^b^	0.001
IgG+	6.0 ± 0.9 ^a^	3.4 ± 0.2 ^b^	5.3 ± 0.5 ^ab^	0.024
IgM+	38.7 ± 1.9	32.1 ± 2.9	37.4 ± 1.6	0.335

FC, free choline; GPC, glycerophosphocholine; LysoPC, lysophosphatidylcholine; PC, phosphatidylcholine. Values are presented as mean ± SEM. *p* value of the main effect of diet analyzed by one-way ANOVA. Multiple comparisons between diet groups have been performed with Duncan adjustment. Means within a row that do not share a common superscript letters a, b are significantly different (*p* < 0.05). The following phenotypes not shown in table were not significantly different among groups (mean ± SEM, % of total cells, n = 18); CD4+CD25+, 7.8 ± 0.9; CD4+CD27+, 62.3 ± 1.5; CD4+CD28+, 70.1 ± 3.6; CD8+CD25+, 4.0 ± 0.4; CD8+CD27+, 66.3 ± 2.5; CD8+28+, 65.1 ± 4.0.

## Supplementary Materials

The following are available online at www.mdpi.com/2072-6643/9/7/713/s1, Table S1: Fatty acid composition of experimental diets; Table S2: Total choline content and relative contribution of the different forms of choline in the stomach contents of offspring at 3 weeks of age from lactating dams fed Control (100% FC), Mixed Choline (MC; 50% PC, 25% FC, 25% GPC) or High GPC (HGPC; 75% GPC, 12.5% PC, 12.5% FC) diets; Table S3: Total number of T and B lymphocyte populations in the spleen and mesenteric lymph nodes of lactating dams fed Control (100% FC), Mixed Choline (MC; 50% PC, 25% FC, 25% GPC) or High GPC (HGPC; 75% GPC, 12.5% PC, 12.5% FC) diets; Table S4: Cytokine production by mesenteric lymphocytes after ex vivo stimulation with LPS from lactating dams fed Control (100% FC), Mixed Choline (MC; 50% PC, 25% FC, 25% GPC) or High GPC (HGPC; 75% GPC, 12.5% PC, 12.5% FC) diets; Figure S1: IL-2 production by splenocytes after ex vivo stimulation with Concanavalin A (ConA) from lactating dams fed Control (100% FC), Mixed Choline (MC; 50% PC, 25% FC, 25% GPC) or High GPC (HGPC; 75% GPC, 12.5% PC, 12.5% FC) diets.
