# Supplementary material for: Feeding a Mixture of Choline Forms during Lactation Improves Offspring Growth and Maternal Lymphocyte Response to Ex Vivo Immune Challenges
Source: Nutrients. 2017 Jul 7;9(7):713. doi: 10.3390/nu9070713 (PMC5537828; doi:10.3390/nu9070713)

nutrients

MDPI

Table S1. Fatty acid composition of experimental diets

Fatty acid	Control	MC	HGPC
	g/100 g of total fatty acids		
C16:0	8.5	8.5	8.0
C16:1 n9	0.2	0.2	0.2
C18:0	24.5	26.3	24.8
C18:1 n9	31.8	31.4	32.4
C18:2 n6	29.3	28.3	28.9
C20:0	1.0	0.9	0.9
C18:3 n3 (ALA)	2.8	3.0	3.3
C20:3 n6	0.3	0.3	0.3
C20:4 n6 (AA)	0.3	0.3	0.3
C22:6 n3 (DHA)	0.3	0.3	0.3
Other fatty acids[2]	1.9	1.8	2.1
Total SFA	33.1	34.9	32.9
Total PUFA	33.2	31.9	32.7
Total n-6	29.5	28.6	29.2
Total n-3	3.6	3.3	3.6
Total MUFA	31.8	31.4	32.4
n-6/n-3	8.1	8.8	8.2
PUFA/SFA	1.0	0.9	1.0

[1] Analysis of the fat mixture added to experimental diets determined by gas liquid chromatography; AA, arachidonic acid; ALA, α-linolenic acid; DHA, docosahexaenoic acid; MUFA, monounsaturated fatty acids; n, omega; PUFA, polyunsaturated fatty acids; SFA, saturated fatty acids;

[2] other fatty acids refer to fatty acids that contributed for less than 0.2% in the diet which included trace of 10:0, 12:0, 14:0, 15:0, 18:1c11, 20:2n-6, 20:5n-3, 22:0, 22:4n-6 and 22:5n-3.

Table S2. Total choline content and relative contribution of the different forms of choline in the stomach contents of offspring at 3 weeks of age from lactating dams fed Free Choline (FC; 100% FC), Mixed Choline (MC; 50% PC, 25% FC, 25% GPC) or High GPC (HGPC; 75% GPC, 12.5% PC, 12.5% FC) diets

	**FC (n=6)**	**MC (n=6)**	**HGPC (n=6)**	***P value***
**Total choline content (mg/100g)**	19.6 ± 1.5	17.9 ± 1.3	20.3 ± 2.6	0.657
**Contribution to total choline (%)**				
Free choline	19.3 ± 3.3[a]	12.9 ± 2.2[a]	5.8 ± 1.3[b]	0.002
LysoPC	11.7 ± 1.9	17.5 ± 3.4	13.3 ± 2.1	0.420
PC	6.4 ± 0.9[b]	18.2 ± 5.3[a]	9.4 ± 0.7[ab]	0.006
GPC	32.0 ± 4.4[b]	41.7 ± 4.8[ab]	53.5 ± 2.2[a]	0.005
Phosphocholine	18.7 ± 3.4[a]	7.1 ± 1.7[b]	9.9 ± 2.7[b]	0.022
Sphingomyelin	9.1 ± 1.3	6.1 ± 1.8	8.1 ± 1.1	0.326

GPC, glycerophosphocholine; LysoPC, lysophosphatidylcholine; PC, phosphatidylcholine
P value of the main effect of diet analyzed by one-way ANOVA. Multiple comparisons between diet groups have been performed with Duncan adjustment.

Table S3. Total number of T and B lymphocyte populations in the spleen and mesenteric lymph nodes of lactating dams fed Control (100% FC), Mixed Choline (MC; 50% PC, 25% FC, 25% GPC) or High GPC (HGPC; 75% GPC, 12.5% PC, 12.5% FC) diets

Cell phenotype	Control (n=6)	MC (n=6)	HGPC (n=6)	*P value*
**Total number of immune cells (x10^6) in spleen[1]**				
Total CD3+ (T cell)	87.6 ± 4.2	76.5 ± 5.5	97.5 ± 13.4	0.266
CD3+CD4+	51.8 ± 2.2	43.4 ± 3.0	49.0 ± 6.4	0.389
CD3+CD8+	30.5 ± 1.7	24.5 ± 2.0	34.3 ± 3.9	0.064
Total CD45RA+ (B cells)	62.7 ± 2.8	71.8 ± 5.4	91.0 ± 12.8	0.075
**Total number of immune cells (x10^6) in mesenteric lymph nodes[1]**				
Total CD3+ (T cell)	14.2 ± 1.6	19.0 ± 1.8	15.2 ± 2.0	0.189
CD3+CD4+	9.8 ± 1.1	13.3 ± 1.4	10.0 ± 1.4	0.131
CD3+CD8+	3.7 ± 0.5	4.9 ± 0.6	3.5 ± 0.8	0.277
Total CD45RA+ (B cells)	15.1 ± 8.1	11.5 ± 0.8	8.4 ± 1.0	0.620

GPC, glycerophosphocholine; LysoPC, lysophosphatidylcholine; PC, phosphatidylcholine

[1]The total number of immune cells (x10^6) in each tissue (spleen or mesenteric lymph nodes) was calculated by multiplying the percentage of the immune cell phenotype by the total number of splenocytes (or mesenteric lymphocytes) isolated (x10^6) /100.

Values are presented as mean ± SEM. *P* value of the main effect of diet analyzed by one-way ANOVA. Multiple comparisons between diet groups have been performed with Duncan adjustment.

Table S4. Cytokine production by mesenteric lymphocytes after *ex vivo* stimulation with LPS from lactating dams fed Free Choline (FC; 100% FC), Mixed Choline (MC; 50% PC, 25% FC, 25% GPC) or High GPC (HGPC; 75% GPC, 12.5% PC, 12.5% FC) diets

	FC (n=6)	MC (n=6)	HGPC (n=6)	*P value*
IL-1-β	107 ± 21	147 ± 14	97 ± 15	0.070
IL-6	111 ± 10	100 ± 9	90 ± 37	0.481
IL-10	128 ± 13	171 ± 21	122 ± 20	0.249
TNF-α	114 ± 20	152 ± 12	114 ± 16	0.172

IL, interleukin; LPS, lipopolysaccharide; TNF, tumor necrosis factor

Values are presented as mean ± SEM. *P* value of the main effect of diet analyzed by one-way ANOVA. Multiple comparisons between diet groups have been performed with Duncan adjustment.

Figure S1. IL-2 production by splenocytes after *ex vivo* stimulation with Concanavalin A (ConA) from lactating dams fed Control (100% FC), Mixed Choline (MC; 50% PC, 25% FC, 25% GPC) or High GPC (HGPC; 75% GPC, 12.5% PC, 12.5% FC) diets

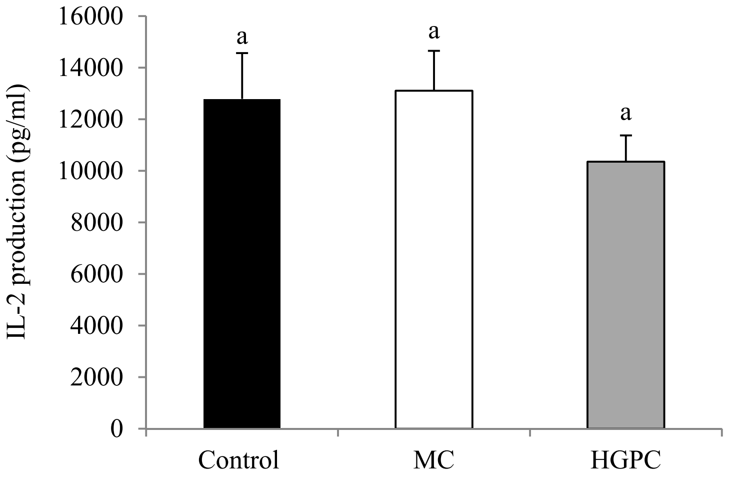

Supplement: Supplementary file 1 [file nutrients-09-00713-s001.zip › nutrients-194542-supplementary.pdf]
